# A Summary of Heat-Flux Sensor Calibration Data

**DOI:** 10.6028/jres.110.009

**Published:** 2005-04-01

**Authors:** A. V. Murthy, G. T. Fraser, D. P. DeWitt

**Affiliations:** Aero-Tech, Inc., Hampton, VA; National Institute of Standards and Technology, Gaithersburg, MD 20899-8441

**Keywords:** calibration, heat flux, sensors, transfer technique

## Abstract

This paper presents a statistical evaluation of the responsivity data on a number of heat-flux sensors, calibrated using an electrical substitution radiometer as a transfer standard up to 5 W·cm^−2^. The sensors, furnished by the customers, were of circular-foil or thermopile type. Comparison of the NIST and the customer measured responsivity values showed that the measurements agree within 3 % for more than half the number of sensors tested, so far. Considering the variation in the customer calibration techniques and the wide measuring range of the sensors used in the calibration, the agreement is encouraging.

## 1. Introduction

The Optical Technology Division (OTD) at the National Institute of Standards and Technology (NIST) has been developing techniques to calibrate heat-flux sensors using thermal radiation from high-temperature blackbodies. Past calibrations at other laboratories and round-robin experiments demonstrated large differences in the measured responsivity of these sensors. The objective of the NIST program was to address this unacceptable discrepancy by developing traceable calibration techniques [1]. As an outcome of this effort, the OTD started offering a heat-flux sensor calibration service. The calibration range was up to 5 W·cm^−2^, referenced to a transfer-standard electrical substitution radiometer. Reference [2] gives a description of the transfer calibration method.

Recently, some customers expressed concern about the NIST transfer technique because of the large differences in the measured responsivity, as high as 10 % to 15 %, between their measurements and the NIST calibration results. The disagreement in measured responsivity values between different laboratories has been a continuing problem [3] in heat-flux sensor calibration. Large uncertainty in the measurements to some extent is a contributing factor for the observed differences. However, the 10 % to 15 % differences reported by some customers were even larger than the reported measurement uncertainties. Consequently, heat-flux sensor calibrations particularly at high flux levels, has been a major topic of discussion at the American Society of Mechanical Engineers (ASME) and American Society for Testing and Materials International (ASTM) committees in recent years.

To resolve the discrepancies in the calibrations and to contribute to a proper understanding of factors involved in heat-flux sensor calibration, OTD initiated several studies. As a part of this activity, OTD offered to perform a blind check-calibration of sensors for the heat-flux measurement community. The objective was to understand whether the large differences in calibration were widespread or just limited to a few isolated cases. The response to the check-calibration offer was overwhelming, resulting in the calibration of a number of sensors for various organizations, both government and private. This report presents results of the OTD calibrations of the sensors and compares the measured responsivities with calibration data furnished by the participating organizations.

## 2. Transfer Calibration Procedure

The OTD transfer calibration technique works on the principle of electrical substitution radiometry. The reference standard is a room-temperature electrical substitution radiometer (ESR). The ESR calibration is traceable to the High Accuracy Cryogenic Radiometer (HACR), the U.S. primary standard for optical radiation power [4], through a chain of calibrations.

**Fig. 1:**
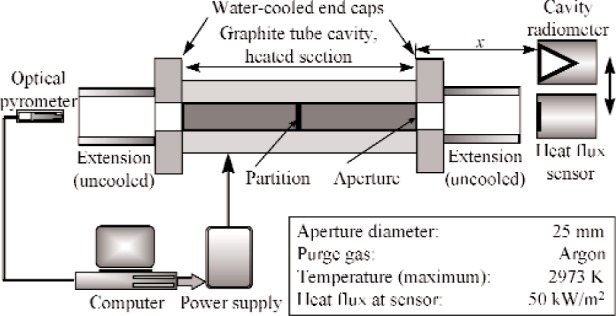
Schematic layout of the NIST 25 mm Variable-Temperature Blackbody.

The radiant source for the calibration is a 25 mm diameter dual-cavity variable temperature blackbody (VTBB) mounted on a computer-controlled horizontal translation stage. Figure 1 shows a schematic layout of the VTBB apparatus and the calibration scheme. The direct resistance heating of the VTBB graphite tube element using large AC currents at low voltages allows quick heating and cooling of the cavity. An optical pyrometer measures the VTBB cavity-partition temperature by sensing radiation from one end of the cavity. A PID controller regulates the power supply to maintain the partition temperature to within ±0.1 K of the set value. The VTBB operating temperature range is from 993 K to 2973 K.

The reference standard ESR is water-cooled and is suitable for continuous operation. During calibration, the test heat-flux sensor and the reference ESR are located outside the blackbody exit in a test plane at a fixed distance from the blackbody aperture. Different calibration ranges are possible by a combination of the sensor location and the VTBB temperature. The distance between the blackbody exit and the test plane is about 12.5 mm for calibrations up to 50 kW·m^−2^. For calibrations in the lower ranges of 25 kW·m^−2^ and 10 kW·m^−2^, the corresponding distances are about 62 mm and 140 mm, respectively.

After stabilization of the temperature to the set value, the VTBB is located in front of the reference ESR and the test sensor, sequentially. The output signals from the instruments are recorded for the test duration in approximately 0.4 s intervals. The sensor responsivity is calculated by a linear regression fit to the sensor signal (mV) data for different values of incident flux (W·cm^−2^) as measured by the radiometer. The measured responsivity of the sensor is expressed in mV·kW^−1^·m^2^ or mV·W^−1^·cm^2^. The regression curve fit for the data is generally linear with regression coefficients close to unity. The relative expanded total uncertainty [5] in the measured responsivity is about 2 % for a coverage factor of *k* = 2.

## 3. Intercomparison Results

The check-calibration comprised of measuring the responsivities of a total number of twelve sensors received from the participating organizations. The sensors calibrated were of thermopile (Schmidt-Boelter) and circular-foil (Gardon) types, which are sensitive to total flux, including both radiative and convective components. The design range of the sensors varied from about 5 W·cm^−2^ to 550 W·cm^−2^. The transfer calibration range was up to 5 W·cm^−2^, which covered only a fraction of the design range of some of the sensors. However, the limited range calibrations of these sensors provide useful data in statistical evaluation of the differences in the measured responsivities.

The check-calibration program covered a period of about 1 year. The transfer technique procedure included the calibration of a reference sensor before measurements on a customer sensor to monitor the long-term repeatability of the procedure. The reference sensor is of Schmidt-Boelter type with a design range of 10 W·cm^−2^. The responsivity value of the reference sensor is 1.191 mV·W^−1^·cm^2^ with a standard deviation of 0.7 % as determined from several calibrations over a period of more than 5 years. Table 1 summarizes the results of the OTD transfer calibration results along with the responsivity data received from the participants. Since the objective is to make a statistical assessment, the names of the individual participating organizations have not been identified. The responsivity values shown refer to the incident flux and the differences refer to deviation from the OTD measured value. The expanded uncertainty (*k* = 2) in OTD measurements varied from 2 % to 2.4 %. However, the corresponding uncertainty data for the participant’s furnished values were not available.

## 4. Discussion

The inter-comparison study performed is the equivalent of a reverse round-robin calibration. The responsivity values of the sensors measured at NIST correspond to using identical experimental setup and instrumentation. The only major variable is the sensor. Therefore, the results of this study have the broader implication of assessing how the OTD transfer technique stands in relation to other techniques. Except for two sensors, which showed deviations of more than 8 %, all the other sensors show a favorable comparison. The calibration methods used by the participating organizations included blackbodies or radiating panels to irradiate the sensors, and the heat-flux at the sensor was calculated by radiometric principles or reference standard calorimeters. However, in most of the cases the view-angle was large in contrast to the narrow view-angle used in the NIST calibrations. Considering the wide variation in the calibration techniques, sensor types and the design ranges, the favorable comparison observed in this study is encouraging.

**Fig. 2:**
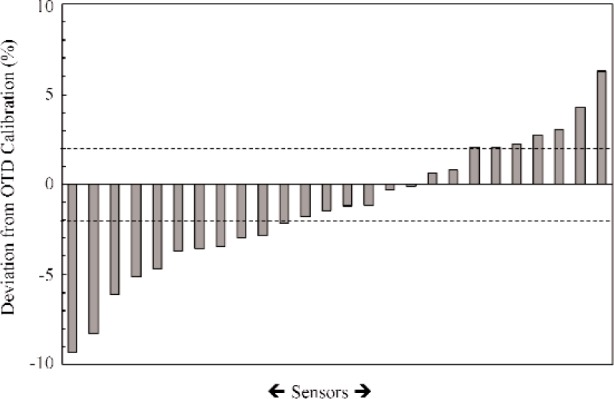
Difference in sensor responsivity between the manufacturer/organization and the OTD calibrations (includes data from both check-calibration and calibration services).

**Fig. 3:**
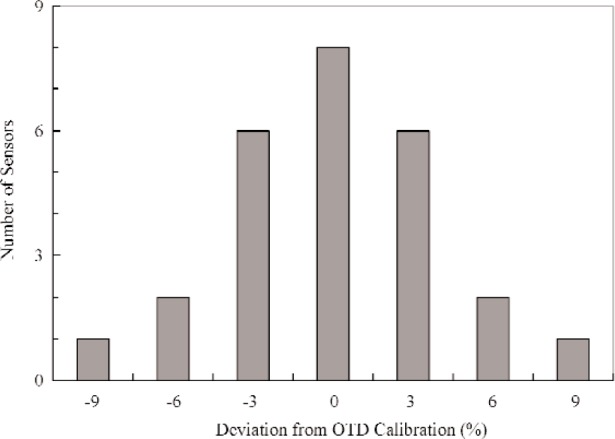
Histogram plot of responsivity deviation from OTD calibrations.

In addition to the check-calibrations discussed above, several other calibrations on heat-flux sensors are in progress on a continuous basis. Figure 2 presents a broader comparison of the responsivities of most of the sensors calibrated so far using the transfer technique. The OTD measurements and the responsivity value furnished by the participating organizations agree within 2 % for about half the number of sensors calibrated, so far. The histogram plot shown in Fig. 3 is close to a Gaussian distribution with fewer sensors showing large deviations from the OTD calibration.

The check-calibration results demonstrate that the calibrations from different organizations and the OTD measurements agree reasonably well. However, what level of deviation is acceptable depends on the end application. A proposed ISO (International Standards Organization) standard [6] specifies an expanded uncertainty of 3 % based on a 95 % confidence level. A previous round-robin calibration by the Federal Aviation Administration had shown larger deviations [3]. The current comparison demonstrates a considerably improved agreement between different calibration methods. A recent round robin [7] between various fire testing laboratories and primary testing laboratories shows similar agreement as the present study. However, the data for the present study comprises a larger number of sensors and more varied calibration methods.

Heat-flux is essentially the equivalent of total irradiance in radiometric terms. The uncertainty in heat-flux sensor calibration is rather large and often inevitable when compared to conventional radiometric measurements involving small spectral irradiance levels. The reasons for the large uncertainty are several. To achieve high flux levels, the sensor location during calibration is close to the radiant source, which results in wider view angles. Therefore, the sensor response depends on non-uniformity in radiation emitted by the different regions of the source and on the angular response of the sensor. Other modes of heat-transfer, particularly convection, can contribute significantly to the total flux at low and moderate irradiance. These factors, along with other variables specific to the particular calibration setup, influence the final measurement uncertainty. According to Moffat [8], uncertainty estimates for a convective environment would be about ±2 % repeatability, ±5 % total uncertainty in calibration, and ±10 % total uncertainty in the application measurements.

For a majority of sensors, the difference is between 2 % and 3 %. This spread represents a situation where several effects, often difficult to control, influence the calibration in all of the methods.

## 4. Concluding Remarks

A review of responsivity measurements performed on several heat-flux sensors shows that the flux-based transfer calibration and the source-based methods often employed by other organizations compare favorably. The agreement between various methods is within 3 % for most of the sensors. Thus, the cases or approaches giving larger deviations need careful evaluation to determine the technical reasons leading to significantly larger discrepancies. The generally good agreement of the responsivities between different techniques reinforces the confidence that the flux transfer calibration based on traceable electrical substitution radiometry can serve as a baseline measurement for calibrating sensors beyond the present transfer calibration limits. The baseline measurement up to 5 W·cm^−2^ serves as a reference to evaluate calibration of the sensor requiring source-based measurements.

## Figures and Tables

**Table 1:** Transfer calibration of heat-flux sensors up to 5 W·cm^−2^

Sensor no.	Responsivity (mV·W^−1^·cm^2^)		
	Customer	OTD	Difference
S01	3.102	3.010	3.1 %
S02	3.324	3.236	2.7 %
S03	2.950	2.891	2.0 %
S04	0.032	0.034	−3.6 %
S05	0.013	0.013	−3.0 %
S06	1.133	1.158	−2.2 %
S07	0.871	0.852	2.2 %
S08	0.520	0.540	−3.7 %
S09	0.484	0.498	−2.8 %
S10	0.793	0.787	0.8 %
S11	0.175	0.193	−9.3 %
S12	0.589	0.642	−8.3 %
